# A Typical Presentation of an Atypical Condition: Hepatic Angiosarcoma With Peritoneal Bleed

**DOI:** 10.7759/cureus.37455

**Published:** 2023-04-11

**Authors:** Soha Afzal, Chintan Patel, Santosh Kagathur, Craig E Cole

**Affiliations:** 1 Internal Medicine, Michigan State University, McLaren Greater Lansing, Lansing, USA; 2 Hematology-Oncology, Michigan State University, Lansing, USA; 3 Hematology and Oncology, Michigan State University, Breslin Cancer Center, Lansing, USA

**Keywords:** hepatology, gastroenterology, oncology, palliative care, hepatic angiosarcoma

## Abstract

Hepatic angiosarcoma (HA) is a rare primary malignancy of hepatic endothelial and fibroblastic vascular tissue origin. Patients typically present with vague constitutional symptoms of fatigue, weight loss, abdominal pain, and ascites. Hemoperitoneum is a frequent clinical manifestation of HA associated with higher mortality and is underrecognized. Here, we report the case of a patient with HA that was complicated by a peritoneal bleed, its management, and associated poor prognosis.

## Introduction

Hepatic angiosarcoma (HA) is a rare malignancy that originates from endothelial and fibroblastic vascular tissue of the liver. It represents up to 2% of primary hepatic malignancies [1]. The presentation usually occurs between ages 60 and 70 and carries an adverse prognosis with overall survival of approximately two years after diagnosis [2]. Due to its highly vascular structure, hemoperitoneum is a frequent finding in these individuals. History can be crucial in diagnosis as patients with HA often have some occupational exposures that include vinyl chloride (associated with *TP53 *mutations), radiocontrast material (associated with *KRAS-2* mutation), oral contraceptive, and androgenic steroid use [3,4]. Here, we report a case of diagnosis, management, and potential prognostication factors for HA in an older patient who presented with nausea, vomiting, and abdominal pain.

## Case presentation

An 81-year-old male presented to a community hospital with nausea, vomiting, and diarrhea while at work. He had a medical history of hypertension, hyperlipidemia, vertigo, and obstructive sleep apnea. He experienced nausea for around four hours before his arrival and an episode of vomiting along with non-bloody diarrhea. Associated symptoms included sharp and non-radiating lower abdominal pain of a few weeks duration with acute worsening on the day of presentation. He denied any other symptoms. The patient reported no occupational exposure to toxic chemicals or vapors such as vinyl chloride monomer or radium. He reported a history of occasional alcohol use but had not consumed any alcohol since the age of 50 years.

In the emergency department (ED), he was noted to be hemodynamically stable. Lab work showed macrocytic anemia with a hemoglobin level of 11.7 g/dL, mean corpuscular volume (MCV) of 103.4 fL, normal renal panel, alanine aminotransaminase (ALT) level of 67 U/L, aspartate aminotransferase (AST) level of 60 U/L, alkaline phosphatase level of 195 U/L, and lactic acidosis of 2.2 mmol/L (Table 1).

**Table 1:** Pertinent laboratory values from the initial and subsequent presentations. MCV: mean corpuscular volume; ALT: alanine aminotransaminase; AST: aspartate aminotransferase; ALP: alkaline phosphatase

	Initial presentation	Subsequent presentation	Normal values
Hemoglobin	11.7 g/dL	9.7 g/dL	14.0–17.0 g/dL
MCV	103.4 fL	105.2 fL	84.0–96.0 fL
ALT	67 U/L	51 U/L	12–78 U/L
AST	60 U/L	59 U/L	15–37 U/L
ALP	195 U/L	271 U/L	46–116 U/L
Lactic acid, venous	2.2 mmol/L	2.8 mmol/L	0.4–2.0 mmol/L

**Figure 1:**
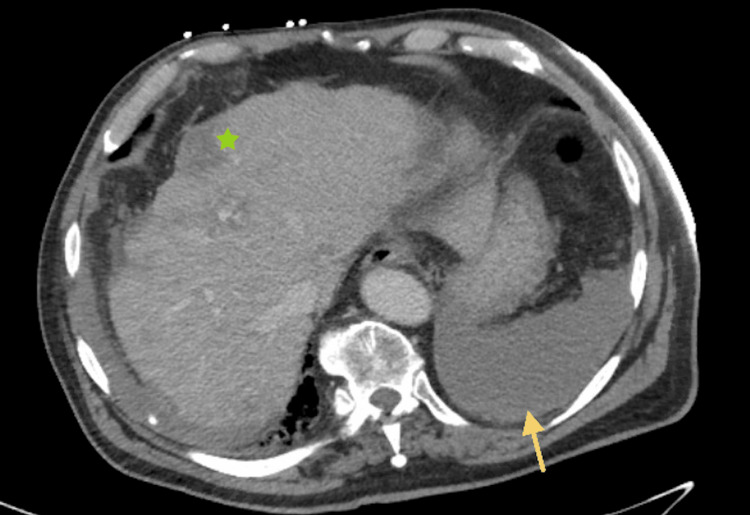
Moderate amount of fluid resembling hemoperitoneum (yellow arrow). Cirrhosis with multiple ill-defined liver lesions abutting the liver capsule (green star).

On admission, a computed tomography (CT) image with contrast of the abdomen and pelvis showed a moderate amount of intermediate-density fluid in the peritoneal cavity suggesting either hemoperitoneum or purulent peritonitis. Other notable findings included a cirrhotic liver with numerous variably sized ill-defined liver lesions, many of which were large and abutting the liver capsule raising suspicion for malignancy with a possible hemorrhage (Figure 1).

**Figure 2:**
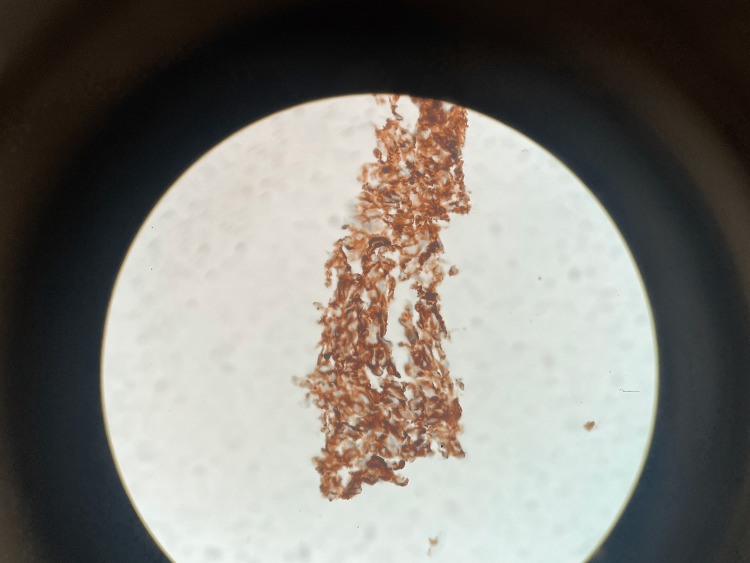
Liver biopsy sample positive for CD31.

His hospital course consisted of a CT-guided liver biopsy and a workup completed with the cardiology team. The gastroenterology and general surgery teams were also consulted and completed a laboratory evaluation that resulted in alpha-fetoprotein, cancer antigen 19-9, and carcinoembryonic antigen levels being within normal limits as well as negative viral hepatitis titers. Pathology from the liver biopsy was consistent with a poorly differentiated tumor with focal areas of blood vessels lined by spindle cells. The blood vessels were lined by hyperchromatic, atypical, spindle-shaped nuclei projecting into vascular spaces within the sinusoids. Immunohistochemical (IHC) staining was positive for CD31 (Figure 2) and Factor VIII-related antigen and negative for CAM 5.2, MART-1, Glypican 3, thyroid transcription factor 1, CD43, and SOX10. D240 staining was inconclusive.

The initial impression suggested angiosarcoma. Due to the inability to perform additional immunostains, the specimen was sent to a tertiary center for a second opinion. The diagnosis of angiosarcoma was confirmed with repeat positivity IHC staining for CD31 and Factor VIII-related antigen. He was discharged home with a referral to the hematology/oncology team to establish care for the management of his newly diagnosed HA.

A month later, he returned to the hospital with similar complaints of intense generalized abdominal pain with associated nausea and diminished appetite along with unintentional weight loss. The ED course consisted of a drop in his blood pressure, the lowest of which was noted at 94/78 mmHg, and macrocytic anemia with a downtrend of hemoglobin to 9.7 g/dL and MCV of 105.2 fL (Table 1).

**Figure 3:**
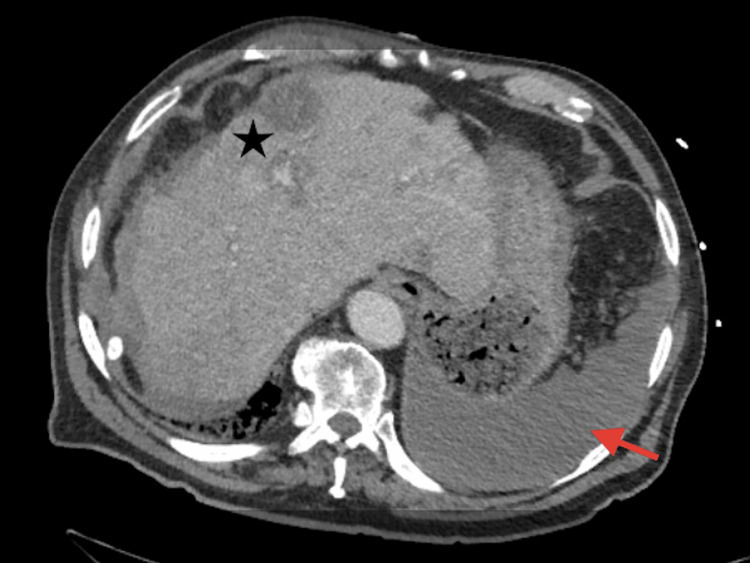
Moderate-volume hyperattenuating ascites mildly increased from the previous examination. Findings indicating hemorrhage (red arrow) and similar hepatic lesions (black star).

A repeat CT abdomen and pelvis with contrast showed moderate ascites along with numerous hepatic lesions and hyperattenuating fluid in the perihepatic region raising concern for hemorrhage (Figure 3).

Given these findings, he underwent celiac, hepatic, and selective segmental hepatic angiography with embolization of the right hepatic lobe by the interventional radiology team to control the hemorrhage. The patient was then discharged home in stable condition. The next couple of months consisted of intermittent episodes of uncontrolled, worsening abdominal pain. He experienced a decline in his performance status to the point where he was unable to care for himself or partake in his activities of daily living. Given this decline, he was deemed not an ideal candidate for systemic therapy by his oncologist. He was eventually transitioned to hospice care and passed away a few weeks later.

## Discussion

HA is a rare liver malignancy originating from endothelial tissue [1]. With around 200 patients identified in the United States, HA primarily occurs in ages 60 to 70 years [2]. Around 75% of tumors have no known etiology, but the most common etiologies include exposure to vinyl chloride monomer, thorium dioxide, androgenic steroid use, and radium [3]. In this malignancy, vinyl chloride has been found to cause *TP53 *mutation, and thorium dioxide can lead to *KRAS-2* mutations. Both of these mutations have been found in cancer cells. Histologically, spindle-shaped and polyhedral cells are found making vascular channels. Cells stain for CD31, CD34, Ulex europaeus agglutinin I, and Factor VIII-related antigen immunohistochemical markers [4]. Patients usually present with non-specific findings such as abdominal pain, jaundice, fatigue, weight loss, and ascites.

A complication of HA has been noted to be peritoneal bleeding [5] which occurs in 17% to 27% of individuals [6,7]. In a 2003 study evaluating the clinical manifestations of primary HA, the presence of spontaneous hemoperitoneum was noted in two out of five patients with less than 45-day survival [8]. When a mass ruptures, this triggers spillage of tumor cells and diffuses sarcomatosis into the peritoneal cavity. This leads to angiosarcomatous seeding lesions being formed, causing brisk hemorrhage, which is seemingly the leading cause of death in this patient population [9]. Our patient died around 42 days after his initial presentation of acute abdominal bleeding. Therefore, it is important to have a high index of suspicion for HA in a patient who may present with new liver masses and a spontaneous hemoperitoneum as it portends an adverse prognosis. This case also highlights the importance of early goals of care conversation with individuals who carry this diagnosis and particularly when they are associated with hemoperitoneum.

It has been recognized that patients diagnosed at an earlier stage (stage Ib) and treated with surgery with or without transarterial chemoembolization (TACE) tend to have favorable outcomes with a median overall survival that can extend beyond five years [10]. On the contrary, in advanced stages, the median survival is close to six months at best with only 3% of patients living beyond two years [9]. The low prevalence of HA likely led to the paucity of prospective clinical trials, which has made it difficult to establish guideline-directed therapies.

Some studies from the 1980s have shown objective improvement in disease with the use of intravenous adriamycin 60 mg/m^2^ for three to four weeks with the possible addition of Cytoxan and methotrexate. Broad-spectrum tyrosine-kinase inhibitors targeting vascular endothelial growth factor receptors such as sorafenib and bevacizumab have shown advances in stable disease. Median progression-free survival was noted to be up to eight months in one group with angiosarcoma treated with sorafenib; however, many of these studies were not specific to HA. Evaluations of antiangiogenic molecules in the setting of angiosarcoma are ongoing [11]. Liver transplantation is not indicated due to high rates of recurrence and HA is considered to be radioresistant [8,12]. Thus, it is prudent to have early goals of care discussions. In our patient, the assistance of the palliative care and hospice teams was necessary for his comfort.

Side effects of conventional chemotherapeutic agents can be quite toxic. Moreover, it may not be the best option for patients with advanced HA as the benefits of aggressive therapy in such patients are unclear [10]. Incurable cancer can cause immense psychosocial and physiologic stress on patients. Incorporation of goals of care discussion with the palliative team has been shown to decrease anxiety, decrease pain, and provide an overall increase in the comfort and quality of life of these individuals [13,14]. Similarly, the use of hospice care in the last six months of a patient’s life has been shown to provide increased patient satisfaction, pain control, and decreased intensive care unit admissions. Preliminary data suggest that early palliative care incorporation may even extend survival in certain conditions [15]. On a review of the literature, case reports have mentioned detection methods, treatment modalities, and outcomes of HA. However, the importance of early goals of care discussions in this patient population has not been emphasized until now (Table 2).

**Table 2:** Treatment and outcomes of patients with hepatic angiosarcoma from previous case reports.

Author, year	Treatment	Outcomes
Locker et al., 1979 [6]	Case 1: Adriamycin and cyclophosphamide	No evidence of change in tumor status. The patient died after a massive intra-abdominal hemorrhage 13 months after the diagnosis
Locker et al.. 1979 [6]	Case 2: Adriamycin, dimethyl triazo-imidazole carboxamide, cyclophosphamide	The patient died during hospital admission for hepatic coma one year after diagnosis. Additionally, 3,000 cc of bloody ascites was noted on autopsy
Locker et al., 1979 [6]	Case 3: Adriamycin	The patient died around six months after diagnosis at a hospital near his home
Locker et al., 1979 [6]	Case 4: Adriamycin	The patient died around a year after diagnosis. Evidence of metastasis was noted on autopsy
Lee et al., 2008 [9]	Hepatic resection	The patient died of recurrent bleeding and disseminated intravascular coagulation around a month after the initial presentation
Chien et al., 2012 [16]	Hepatic resection	There was no local recurrence or distant metastasis four months after the procedure
Chen et al., 2016 [17]	Hepatic resection	The patient died within one month of diagnosis. The patient went into cardiac arrest after undetectable vital signs on postoperative day three
Masanori et al., 2017 [18]	Hepatic resection	The patient died 13 days after the presentation from massive bleeding
2018 [5]	Gemcitabine	The patient died seven months after diagnosis from disease complications
Flabouris et al., 2021 [19]	No documented treatment	The patient died three months after her initial presentation from hypotension and significant anemia
Manh Hung et al., 2022 [20]	Doxorubicin	The patient died in the sixth month of treatment after shortness of breath and severe abdominal pain
Kashiwadate et al., 2022 [21]	Hepatic resection	There was evidence of recurrent disease. The patient requested supportive care after recurrence was confirmed and died over a month after the presentation

## Conclusions

HA is a rare and rapidly progressing primary malignancy of the liver. Patients present with non-specific symptoms that frequently result in diagnostic delays. Hemoperitoneum is a common complication of this cancer and may serve as a sign of disease progression that heralds poor prognosis. There are currently no guideline-directed therapies given the lack of clinical trials, and treatments are largely based on case series at best. This case describes the importance of considering HA in the differential of a patient with liver masses and hemoperitoneum. It also emphasizes the importance of engaging the palliative care team early in the course of illness as it results in an overall better quality of life.
